# Effects of Obstructive Sleep Apnea Syndrome and Medical Comorbidities on Language Abilities

**DOI:** 10.3389/fneur.2021.721334

**Published:** 2021-09-22

**Authors:** Konstantinos Makanikas, Georgia Andreou, Panagiotis Simos, Efstathia Chartomatsidou

**Affiliations:** ^1^Department of Special Education, University of Thessaly, Argonafton & Filellinon, Volos, Greece; ^2^School of Medicine, University of Crete, Heraklion, Greece; ^3^Computational Biomedicine Laboratory, Institute of Computer Science, Foundation for Research and Technology-Hellas, Heraklion, Greece

**Keywords:** obstructive sleep apnea, semantic language, verbal fluency, hypercholesterolemia, hypertension, diabetes, cardiovascular disease, oxygen desaturation

## Abstract

**Objective:** The primary objective of the present cross-sectional study is to evaluate the semantic language abilities of patients with Obstructive Sleep Apnea Syndrome (OSAS) compared to normative data. Secondary objectives are to examine the effects of OSAS comorbidities on language test performance.

**Method:** 118 adult patients suffering from OSAS were assessed using standardized tests (Boston Naming Test, the Peabody Picture Vocabulary Test and the Verbal Fluency Test).

**Results:** Compared to normative standards, the OSAS group (age and education adjusted mean) scored significantly lower on all tests (*p* < 0.01). The OSAS group also included a significantly higher percentage of persons scoring below the 5^th^ percentile of the normative distribution on the four tests (*p* < 0.01). The Apnea/Hypopnea Index, O_2_ Desaturation index, SaO_2_ <85% (min) and SaO_2_ <75% (min) were significantly associated with language test scores (*p* < 0.05). Moreover, higher Apnea–Hypopnea Index score and night-time oxygen desaturation were associated with reduced phonemic and semantic fluency performance only among patients with a history of hypertension and hypercholesterolemia (*p* < 0.05). The moderating effect of diabetes and cardiovascular disease on the association between OSAS severity indices and test scores did not reach significance (*p* > 0.6).

**Conclusions:** Results suggest that the severity of semantic language impairments in patients with OSAS is associated with the severity of the disease and intensified by common medical comorbidities (hypertension and hypercholesterolemia).

## Introduction

Obstructive Sleep Apnea Syndrome (OSAS) is a common sleep-related breathing disorder affecting 5% of the general population (1). OSAS is characterized by periodic complete or partial cessation of breathing while sleeping. These recurrent events of breathing result in fragmented sleep due to increased sleep arousals that terminate the apneic episodes and in recurrent hypoxemia (reductions in hemoglobin oxygen levels) due to upper airway obstruction (2). OSAS has been known to be associated with comorbid diseases such as cardiovascular system diseases and metabolic diseases. Furthermore, patients with OSAS may show reduced performance on a wide range of cognitive functions such as attention, memory and executive function (3–5).

Few studies have examined language abilities in patients with OSAS, and the majority of those studies used only the phonemic and semantic verbal fluency tests, which require initiation and shifting skills, intact semantic memory and effective search processes (6).

For instance, Lim et al. reported verbal fluency scores in the impaired range in 30.4% of severe OSAS patients (7). Ferini-Strambi et al. found that phonemic fluency performance in OSAS patients was significantly poorer than healthy controls. However, this was not observed in the semantic fluency task. Moreover, it has been found that the phonemic fluency test significantly correlated with the duration of oxygen desaturation (8). On the other hand, Twigg et al. found that the performance of patients with moderate OSAS was similar to healthy controls on both semantic and phonemic verbal fluency tests (9). Torelli et al. also found that 16 patients with moderate-severe OSAS performed similarly to a healthy control group on measures of phonemic and semantic verbal fluency (10, 11). However, common non-surgical treatment for OSAS (Continuous Positive Airway Pressure therapy; CPAP) for as little as 15 days has been found to improve semantic verbal fluency performance at least in patients with severe OSAS (7, 8).

The literature is equivocal regarding the relative importance of hypoxemia (12) and sleep fragmentation (7, 13, 14) as causes of neurocognitive deficits in OSAS (15). Importantly, little is known regarding the contribution of comorbidities such as hypertension and diabetes, which are known risk factors for cognitive decline (16), on the severity of cognitive impairment in OSAS. The present study addresses this gap in the literature, by assessing a wide range of language abilities (semantic-category and phonemic verbal fluency, receptive vocabulary and confrontational naming) in a large sample of middle-aged adults with OSAS. In addition, the impact of hypoxemia, fragmented sleep, and medical comorbidities (hypertension, diabetes, hypercholesterolemia, cardiovascular diseases) on language test performance was examined. We hypothesized that patients with OSAS would display reduced performance compared to expectations for age and education level and that there would be a correlational link between OSAS characteristics and language tasks.

## Methods

### Participants

The sample included 118 OSAS patients aged 50–69 years (90 men and 28 women) with 10.35 ± 4.15 years of education (range: 1–20 years). The patients were recruited over a period of 2 years at the Pulmonary Department, University Hospital of Larisa, Greece (see Table 1 for demographic and clinical characteristics of the sample). They were referred for an overnight sleep study to investigate daytime and nocturnal symptoms of sleep disorder, such as daytime somnolence, non-refreshing sleep, insomnia, snoring and other signs of sleep apnea. All patients were native speakers of Greek and resided in central Greece. Inclusion criteria were the following: (a) diagnosis of OSAS as indicated by an Apnea Hypopnea Index (AHI) score >5/h, (b) no previous treatment for apnea, (c) no other diagnosable sleep disorder, (d) absence of global cognitive impairment as indicated by a Mini Mental State Examination score <24 points), and (e) no history of head injury, cerebral ischemia, encephalitis, or psychiatric disorders including Attention Deficit Hyperactivity Disorder (ADHD), alcohol and drug abuse.

**Table 1:** Demographic and clinical characteristics of OSAS patients.

Men	90 (76.3%)
Women (%)	28 (23.7%)
Age	58.40 ± 5.17 (50–69)
Education in years	10.35 ± 4.15 (1–20)
Body Mass Index	32.83 ± 5.79 (24–53)
Hypertension	58 (57.6 %)
Hypercholesterolemia	48 (40.7 %)
Cardiovascular diseases	22 (18.6 %)
Diabetes	15 (12.7 %)
ESS	8.99 ± 4.04 (1–20)
AHI	35.94 ± 22.10 (7.7–94.9)
AI	16.74 ± 19.9 (0–89.2)
HI	19.2 ± 12.01 (2.2–62.3)
Sleep arousal index	45.18 ± 20.45 (15.5–103.7)
Total sleep time (min)	258.08 ± 63.15 (111–410)
TST stage 1 (%)	4.28 ± 2.51 (0–13.1)
TST stage 2 (%)	67.87 ± 10.85 (39.9–97.5)
TST stage 3 + 4 (%)	13.28 ± 8.98 (0–36.7)
TST stage REM (%)	14.56 ± 6.78 (0–30.4)
O_2_ desaturation index >3%	36.49 ± 27.22 (1.3–113)
Desaturation / hour of sleep (min)	15.06 ± 12.82 (0.6–48.8)
SaO_2_ time min <95%	147.17 ± 90.54 (0.6–334.20)
SaO_2_ time min <90%	31.98 ± 54.77 (0–235.20)
SaO_2_ time min <85%	10.46 ± 25.02 (0–165.10)
SaO_2_ time min <80%	3.73 ± 11.77 (0–93.00)
SaO_2_ time min <75%	1.32 ± 5.27 (0–44.10)
Nocturnal SaO_2_ mean sleep	90.49 ± 4.38 (75–97)
Nocturnal SaO_2_ during episodes	89.40 ± 4.21 (75–96)

*Unless otherwise noted all values are mean ± SD with range in parentheses. AHI, Apnea-Hypopnea Index, AI, Apnea Index, HI, Hypopnea Index, ESS, Epworth Sleepiness Scale, TST, Total Sleep Time; SaO_2_, Oxygen saturation*.

The Epworth Sleepiness Scale (ESS) was used to evaluate the presence and severity of daytime sleepiness in daily life. Among OSAS patients, 51.7% scored ≥8 points indicating excessive daytime sleepiness (17). Since high blood pressure, hypercholesterolemia, cardiovascular disease and diabetes are common features of OSAS, patients whose comorbid conditions were well controlled with medications were included. The distribution of patients to OSAS severity grades according to AHI score criteria (2) were as follows: mild OSAS (AHI ≥ 5 but <15; *n* = 36), moderate OSAS (AHI = 15–30; n = 24), and severe OSAS (AHI ≥ 30; *n* = 58).

A comparison group of 174 adults (70.7% men and 29.3% women) aged 50–69 years (mean age = 58.60 [SD = 5.30] years) with 10.80 [SD = 4.40] years of formal education were selected from the Greek normative sample for the language tasks employed in the present study to match the OSAS group on gender, age and education level (*p* > 0.2) (18). All participants in the normative sample were screened for common medical comorbidities, and those reporting diagnosed OSAS or other sleep disorder were not included in the comparison group. The comparison sample was formed in the following steps: (i) Selection of participants with age and education within the corresponding ranges of the patient group, (ii) Selection of 100 random subsamples from this reduced normative group bearing the same male/female ratio as the patient group, (iii) Step ii was repeated 10 times by increasing subsample size in steps of 10 (range: 114 to 204 persons), (iii) The deviation of each subsample from the patient group on age and education, combined, was quantified using the mean square error metric, which served as the criterion for selecting the subsample that most closely matched the patient group on demographics. The study was approved by the University Hospital of Larisa Ethics Committee and all subjects gave their informed consent prior to their participation.

### Materials and Procedure

Neurocognitive testing was conducted in a single session, 1–2 days following the night sleep study, between 9:00 and 14:00. Testing lasted between 30 and 60 min and participants were allowed to take breaks in order to minimize fatigue. The tests were administered in the same order to all participants and by the same examiner.

Confrontation naming was assessed using the Greek short version of the Boston Naming Test (BNT) consisting of monochrome drawings of 45 common objects. Performance on this test requires recognition of the depicted object, retrieval and verbal production of the lexical entity associated with it. Access to associated semantic representations is assumed to take place automatically although, in principle, it is not required for task performance (18). In the original validation study, there was excellent concordance between the short and extended versions of the BNT (*r* = 0.94), and very good internal consistency (Cronbach's α = 0.85) and 2-week test-retest reliability (*r* = 0.83) (18).

Receptive vocabulary (availability of lexical representations for noun, adjective, and verb concepts) was assessed using the Greek adaptation of the Peabody Picture Vocabulary Test-R (PPVT-R) consisting of 32 plates in its short version. Each stimulus plate presents four gray-scale drawings and the examinee is asked to point to the one that best-matches a word spoken by the examiner. Due to the special response requirements of the task, it is reasonable to expect that perceptual organization and decision-making ability may account for a certain amount of individual variability in performance (18). In the original validation study, there was excellent concordance between the short and extended versions of the PPVT (*r* = 0.91) as well as internal consistency (Cronbach's α = 0.84) and two-week test-retest reliability (*r* = 0.86) (18).

Finally, rapid access to stored lexical representations was assessed using two-word generation tasks (6). In the Semantic Verbal Fluency task participants were asked to produce words belonging to each of the following three semantic categories: animals, fruits and items. In the Phonemic Verbal Fluency task participants were asked to produce words beginning with each of the following Greek letters: *X* (Chi), S (Sigma), and *A* (Alpha). Despite some commonalities, these two tasks require different cognitive processes. Adequate semantic fluency requires intact semantic memory stores and effective search processes. In contrast, phonemic fluency is less dependent on memory stores, and more dependent on effective initiation and shifting skills. Both fluency tasks are timed and participants are encouraged to generate words as fast as they can within the allocated time-limit (60 sec per category or letter). Two-week, test-retest reliability of the Phonemic subtest was 0.86 and 0.83 for the semantic subtest in a small sample of cognitively intact elders (*n* = 19, mean age: 66.0, SD = 7.1 years) (19).

Raw scores on language tests were converted to age and education-adjusted standard (z) scores based on normative data (6, 18). Z scores < −1.645 were considered as indicating deficient performance, corresponding to the 5^th^ percentile of the normal distribution of the normative sample.

Nocturnal polysomnography was carried out at the University Hospital of Larisa Sleep Lab from 23:00 to 07:00, employing EEG, electro-oculography, electromyography and electrocardiography for conventional sleep staging by a certified technician. The Alice Respironics v. 2.8.78 software was used for data acquisition. Sleep structure was analyzed through visual scoring, following the standard procedure (20) and the criteria for EEG arousals defined by the American Sleep Disorders Association (21). Respiratory events were scored according to the criteria of the American Academy of Sleep Medicine (2). Thoracoabdominal respiratory movements were recorded by strain gauges, nasal airflow by nasal cannula, snoring by tracheal microphone and oxygen saturation by finger pulse oximeter. A decrease in oxygen saturation of 3% or more from the baseline level was regarded as clinically significant. Episodes of apnea were defined as complete cessations of airflow for 10 s or more, and episodes of hypopnea as decreases in nasal airflow of more than 50%, and lasting for at least 10 s, accompanied by desaturations ≥3% or microarousals.

The following parameters computed from the overnight polysomnographic recordings were considered as objective complementary measures of sleep quantity and quality (total sleep time, percentage of time spent in sleep stages (1 + 2) and (3 + 4), REM sleep, number of awakenings) and night-time blood oxygenation (total number of desaturation per hour of sleep, duration of desaturation per hour of sleep in min, mean arterial oxygen levels during sleep and during AHI episodes, percentage of sleep with SaO_2_ below 75–95%). An overall index of OSAS severity was also computed in the form of the AHI. Night sleep quality was further computed in the form of sleep efficiency (%), sleep arousal, and proportion of sleep in stage 1.

Common medical comorbidities (hypertension, hypercholesterolemia, cardiovascular disease, diabetes) were also recorded (see Table 1). Illness duration was not recorded since obstructive sleep apnea symptoms generally begin insidiously and are often present for years before patients are referred for evaluation.

### Analyses

Data were analyzed using IBM SPSS statistics 21 and Minitab 17.0. Results are given as mean ± standard deviation (SD). After a descriptive analysis of the samples' characteristics (sociodemographic and clinical date) the following analyses were performed: Z scores were calculated for the neuropsychological test for which normative data were available (controlling for gender, age and education). Higher Z scores indicate better performance. Scores below the 5^th^ percentile of the normative distribution were considered as indicating deficient performance.

The degree of deviation of language test scores from the corresponding means of the comparison sample was examined through two-tailed, independent-sample *t*-tests, while differences between the patient and comparison group in the percentage of scores in the deficient range was assessed using the *X*^2^ test of proportions. The nominal alpha level was set to 0.0125 to correct for the four comparisons performed on each test.

The association between polysomnographic parameters and language test scores was assessed through linear multiple regressions. Preliminary analyses indicated significant positive skewness (as indicated by significant Kolmogorov-Smirnov tests) for AI, O_2_ desaturation index, desaturation durations, and average nocturnal and apnea episode saturation. Accordingly, these variables were log-transformed prior to entering into the moderated regression models. In separate models, the additive effect of medical comorbidities on these associations was examined through moderated regression analyses, using PROCESS, a freely available computational tool for SPSS v. 20 (22). Age, gender, education, and Epworth Sleepiness Scale total score were included as covariates in the models. The *p* value in assessing statistical significance was set to 0.05.

## Results

Although average age and education-adjusted scores of OSAS patients were within the normal range (i.e., no more than 1 SD below the population mean), these were significantly lower than the comparison group on all language tests (*p* < 0.002). In particular, mean scores of OSAS patients were Z = −0.44 on BNT, Z = −0.63 on PPVT-R, Z = −0.63 on Semantic verbal fluency and Z = −0.64 on Phonemic verbal fluency. Moreover, the percentage of OSAS patients scoring at least 1.645 SDs below the population mean was 13.6% on semantic verbal fluency, 16.9% on phonemic verbal fluency and 13.6% on PPVT-R. The above-mentioned percentages were significantly higher among patients than the comparison group (*p* < 0.001; see Table 2), where only a small percentage of the participants (i.e., no more than 3.5% of the sample) scored below the 5^th^ percentile of the normative distribution. There was no significant difference on BNT between percentage of OSAS patients (7.6%) and normative participants (4.6%) with deficient scores.

**Table 2:** Performance of OSAS patients and comparison group on language tests.

	**Mean (SD)**			**% below 5** ^**th**^ **%tile**		
**Language test**	**OSAS patients**	**Comparison group**	***P* value^a^**	**OSAS patients**	**Comparison group**	***P* value^b^**
Semantic fluency	−0.63 (0.87)	−0.05 (0.95)	0.0002	13.6	2.9	0.0005
Phonemic fluency	−0.64 (0.99)	0.08 (1.0)	0.001	16.9	3.5	0.0002
BNT	−0.44 (0.89)	0.002 (1.0)	0.002	7.6	4.6	0.1
PPVT-R	−0.63 (0.94)	0.11 (0.89)	0.0001	13.6	3.5	0.0007

a *Two-tailed, independent-sample t-test OSAS patients and the gender-, age-, and education level-matched comparison group on z scores*.

b *Chi-Square test for proportions of OSAS patients scoring in the deficient range (N = 118) as compared to the comparison group (N = 174). BNT, Boston Naming Test, PPVT-R, Peabody Picture Vocabulary Test-Revised*.

Given the relatively small size of the moderate OSAS severity group, the mild (*n* = 36) and moderate severity groups (*n* = 24) were lumped together (in preliminary comparisons the two groups displayed largely comparable language scores, *p* > 0.3). Controlling for demographics and ESS score, the subgroup of patients displaying severe OSAS (*n* = 58) performed marginally lower than the mild/moderate severity subgroup (*n* = 60) on phonemic verbal fluency [z = −0.46, SD = 1.01 vs. z = −0.81, SD = 0.86, respectively; F (1,115) = 3.25, *p* = 0.07]. The severe-OSAS group scored slightly lower than the mild-moderate OSAS group on PPVT-R (z = −0.52, SD = 0.90 vs. z = −0.72, SD = 0.92), BNT (z = −0.38, SD = 0.81 vs. z = −0.49, SD = 0.97), and semantic verbal fluency (z = −0.59, SD = 0.85 vs. z = −0.67, SD = 0.90), but these differences did not approach significance (*p* > 0.2).

The overall effect of each polysomnographic parameter on language scores failed to reach significance (*p* > 0.1) in models that included age, education, gender, and ESS score as covariates. However, in exploratory multiple regression analyses that did not control for ESS, the effect of sleep parameters, related to hypoxemia, on phonemic verbal fluency scores were significant: Apnea/Hypopnea Index (B = −0.83, *p* = 0.025), O_2_ Desaturation index (B = −0.062, p = 0.039), SaO_2_ <85% (min) (B = −0.065, *p* = 0.048), and SaO_2_ <75% (min) (B = −0.329, *p* = −0.038). Objective sleep quantity and quality measures (duration of sleep stages, number of awakenings) did not approach significance (*p* > 0.2).

Finally, moderated regression models were computed to assess the combined effect of sleep disturbances and cardiovascular risk factors on language scores among OSAS patients, controlling for age, education, gender, and ESS score. Results, displayed in Table 3, reveal that the association between OSAS severity (as indexed by AHI and O_2_ DI) and performance on both verbal fluency tests was moderated by hypertension and hypercholesterolemia. Specifically, the negative effect of AHI on both phonemic and semantic verbal fluency test scores reached significance only among OSAS patients who also presented with either comorbid condition. In addition, the negative effect of O_2_ DI on semantic verbal fluency scores was significant only when combined with hypertension and hypercholesterolemia. Interaction terms involving diabetes and cardiovascular disease did not reach significance (*p* > 0.6).

**Table 3:** Unstandardized regression coefficients of OSAS severity and medical comorbidities on verbal fluency test scores.

	**Independent variable**	**Moderator**	**Independent variable x Moderator**	**Conditional effect of sleep index**	
Verbal Fluency test	*AHI*	*Hypertension*	*AHI x Hypertension*	*without Hypertension*	*with Hypertension*
Semantic	−0.012 [−0.08 to.06]	2.51 [−0.42 to 5.46]	−0.18 [−0.31 to −0.05]^*^	0.093 [−0.007 to.19]	−0.09 [−0.17 to −0.01]^**^
Phonemic	−0.08 [−0.14 to −0.023]^**^	1.15 [−2.08 to 4.38]	−0.14 [−0.27 to −0.02]^*^	0.005 [−0.11 to.11]	−0.13 [−0.22 to −0.03]^**^
	*AHI*	*HCL*	*AHI x HCL*	*without HCL*	*with HCL*
Semantic	0.002 [−0.07 to.06]	−1.64 [−4.54 to 1.28]	−0.15 [−0.28 to −0.02]^*^	0.06 [−0.03 to.16]	−0.09 [−0.18 to −0.017]^*^
Phonemic	−0.06 [−0.12 to −0.01]^*^	−2.06 [−5.14 to 1.01]	−0.12 [−0.17 to −0.08]^*^	−0.05 [−0.16 to.02]	−0.10 [−0.20 to −0.001]^*^
	*O_2_ DI*	*Hypertension*	*O_2_ DI x Hypertension*	*without Hypertension*	*with Hypertension*
Semantic	−0.006 [−0.05 to.04]	2.55 [−0.31 to 5.40]	−0.15 [−0.25 to −0.04]^*^	0.08 [−0.01 to.17]	−0.07 [−0.12 to −0.01]^**^
Phonemic	−0.05 [−0.10 to.01]	1.05 [−1.96 to 4.06]	−0.12 [−0.22 to −0.02]^*^	0.02 [−0.05 to.09]	−0.10 [−0.17 to −0.03]^**^
	*O_2_ DI*	*HCL*	*O_2_ DI x HCL*	*without HCL*	*with HCL*
Semantic	−0.01[−0.05 to.05]	−1.71 [−4.44 to 1.10]	−0.10 [−0.18 to −0.01]^*^	0.04 [−0.03 to.10]	−0.07 [−0.13 to −0.02]^*^
Phonemic	−0.05 [−0.10 to.01]	−2.27 [−5.35 to 0.82]	−0.004 [−0.12 to.11]	−0.05 [−0.14 to.01]	−0.04 [−0.17 to.06]

*CI = 95% Confidence Intervals (bias corrected and accelerated based on 5,000 bootstrap samples), after controlling for age, education, gender, and ESS score. AHI, Apnea-Hypopnea Index; O_2_ DI, O_2_ desaturation Index (≥3%), HCL, hypercholesterolemia*.

* *p < 0.05*,

** *p < 0.001*.

## Discussion and Conclusion

The present sample of middle-aged OSAS patients scored significantly lower on language tasks, assessing availability and efficiency of access/retrieval of lexical-semantic representations, than expected based on their demographic characteristics. This finding extends previous reports focusing exclusively on verbal fluency tasks (3) to suggest that performance on untimed lexical-semantic tasks may also be affected in OSAS. Notably, the percentage of patients scoring within the deficient range on verbal fluency tasks (13–17%) was close to that reported in a previous study (7).

The cross-sectional nature of the present study notwithstanding, we found some evidence for a correlational relationship between OSAS characteristics and language performance, which was restricted to verbal fluency tasks. Our findings support earlier conclusions regarding the importance of night-time hypoxemia as a key contributor to verbal fluency deficits in OSAS (10) and failed to demonstrate direct associations between verbal fluency performance and sleep characteristics, such as sleep fragmentation and frequent arousals (13, 14). Importantly, the effects were accounted for by specific insomnia symptoms, namely excessive daytime sleepiness. To the extent that daytime somnolence may affect attention capacity, the present results are consistent with the possibility that hypoxia during sleep could affect daytime vigilance, attention capacity and secondarily reduce performance on timed language tests. It should also be noted that the present study was not designed to address the pathophysiological substrate of language impairment, which may involve a wide range of, potentially interrelated, processes such as microvascular cerebral pathology (3), and changes in systemic hemodynamics and inflammation (23).

Importantly, the degree of the (negative) association between OSAS severity, as indexed by AHI and average night-time O_2_ saturation, and both verbal fluency tasks was moderated by the presence of two common medical comorbidities: hypertension and hypercholesterolemia. Specifically, higher AHI scores and night-time desaturation were associated with reduced phonemic and semantic fluency performance only among patients with a history of each of the two comorbid conditions. These effects persisted after controlling for daytime sleepiness. Although presence of either condition alone did not correlate with verbal fluency performance, hypertension or hypercholesterolemia combined with OSAS appeared to correlate significantly more strongly with reduced verbal fluency.

The present results suggest an additive effect of cardiovascular risk factors and sleep apnea/sleep hypoxia on performance on timed language tests. It is not uncommon to find weak total effects of cardiovascular risk factors on cognitive task performance especially on less demanding tasks that do not depend heavily upon working memory, episodic memory, and attention capacity (24–26). Moreover, although we did not assess the duration and severity of cardiovascular risk factors, a weak effect may be explained by a short duration and low degree of cardiovascular risk factors in the group of the middle-aged patients (27). In addition, there is some evidence of a U-shaped effect of cholesterol levels on cognitive task performance, in which optimal concentrations are associated with higher performance, whereas overly high or low concentrations are associated with lower scores (28). More importantly, hypertension and hypercholesterolemia were well controlled with medications in the great majority of OSAS patients in our study and there is data to suggest that treatment of these risk factors for vascular disease may prevent further cognitive deterioration (29–31).

Performance on both verbal fluency tasks was significantly related to OSAS severity (as indicated by the Apnea/Hypopnea Index) and hypoxemia. Therefore, the putative impact of OSAS on verbal fluency may not be attributed to the disruption of processes shared by the remaining two verbal tasks administered in the present study, namely availability of lexical-semantic representations (as indexed by PPVT-R and BNT) and retrieval of phonological representations (as indexed by BNT). Likely candidate processes accounting for reduced performance in the verbal fluency tasks used in the present study include (a) A general disruption of processing speed given that performance on both verbal fluency tasks is time-dependent, and (b) A more selective disruption of the efficient organization of lexical-semantic networks (as implied by reduced performance on PPVT-R and BNT), affecting efficient retrieval of stored representations, which becomes more pronounced under time-pressure.

It is difficult to speculate on the factors related to previous unsuccessful attempts to establish language impairment in OSAS patients (8, 11). The present results highlight a few relevant factors. First, the effect size of the putative impairment is small, considering that average performance of OSAS patients on all language tasks remained within normal range. Therefore, effects may be statistically undetectable with small sample sizes. Secondly, the impact of OSAS on language abilities may have been obscured by systematic individual differences in patient health characteristics, which were not included in the analyses as explanatory variables. In addition, since obstructive sleep apnea symptoms are often present for years before the syndrome is diagnosed, it is difficult to assess if the chronicity of illness may mediate the relationship with cognitive functioning.

Our study has several limitations. Thus, the participants of the normative group were not assessed for possible obstructive sleep apnea symptoms and therefore latent OSAS patients might have been included in the normative group. However, this misclassification would have led to smaller group differences rather than contributing to false positive results. A second potential limitation concerns the mediating role of attention difficulties in the association between OSAS and language performance. Such difficulties may be related, among other conditions, to (a) undiagnosed ADHD or subclinical ADHD-type symptoms which were not assessed formally in our study, and (b) to the direct effects of OSAS on attention capacity. In this context it is important to note that the duration of the assessment session was kept short by design (40–60 min) in order to prevent fatigue and diminishing motivation (32).

Moreover, to the extent that the common symptom of excessive daytime sleepiness (EDS) may be used as an adverse effect of OSAS on attention capacity, we conducted supplementary analyses to evaluate this possibility. Results showed that scores on the Epworth Sleepiness Scale accounted for very little variance on untimed tests (3.7 and 1.5% on age- and education-adjusted PPVT and BNT scores, respectively), and a modest amount of variance of verbal fluency tests (6.4 and 10.8% on age- and education-adjusted semantic and phonetic verbal fluency tests, respectively). Accordingly, EDS was included as an additional covariate in all the regression analyses reported in the present work. Future studies should address the potential mediating effect of attention capacity on the impact of OSAS on verbal-semantic abilities.

Lastly, the cross-sectional nature of the study does not permit drawing conclusions regarding the long-term effects of OSAS on language performance, which would require a longitudinal, prospective study.

In conclusion, OSAS may affect language abilities, and especially those that involve efficient retrieval of lexical - semantic representations. Overall severity of the disorder and night-time O_2_ levels emerged as the most important correlates of verbal fluency performance. Our data support the clinical value of neurocognitive evaluation in OSAS patients. Moreover, highlight the significance of a history of obstructive sleep apnea when evaluating patients with memory and language deficits. Finally, the finding that the effect of OSAS was moderated by the medical comorbidities of hypertension and hypercholesterolemia, which are common in this disorder, is an excellent demonstration of the complexities of diagnostic work.

## Data Availability Statement

The raw data supporting the conclusions of this article will be made available by the authors, without undue reservation.

## Ethics Statement

The studies involving human participants were reviewed and approved by the University Hospital Committee of Larisa. The patients/participants provided their written informed consent to participate in this study.

## Author Contributions

KM conducted the experiments under the supervision of GA, statistical analysis under the supervision of PS, wrote the experimental part of the manuscript with the help of PS, and wrote the discussion with the help of EC. PS helped with the statistical analysis and tables, corrected/added parts in the discussion. GA wrote the abstract of the manuscript and theoretical part, corrected/added parts in the discussion. EC wrote the bibliography and helped with the discussion. All authors contributed to the article and approved the submitted version.

## Conflict of Interest

The authors declare that the research was conducted in the absence of any commercial or financial relationships that could be construed as a potential conflict of interest.

## Publisher's Note

All claims expressed in this article are solely those of the authors and do not necessarily represent those of their affiliated organizations, or those of the publisher, the editors and the reviewers. Any product that may be evaluated in this article, or claim that may be made by its manufacturer, is not guaranteed or endorsed by the publisher.

## References

[1] Young T Peppard P Gottlieb DJ. Epidemiology of Obstructive Sleep Apnea: A Population Health Perspective. Am J Respir Crit Care Med. (2002) 165:1217–39. 10.1164/rccm.2109080 11991871

[2] American Academy of Sleep Medicine Task Force. Sleep-related breathing disorders in adults: recommendations for syndrome definition and measurement techniques in clinical research. Sleep. (1999) 22:667–89. 10.1093/sleep/22.5.667 10450601

[3] Aloia MS Arnedt JT Davis JD Riggs RL Byrd DE. Neuropsychological sequel of obstructive sleep apnea-hypopnea syndrome: a critical review. J Int Neuropsychol Soc. (2004) 10:772–85. 10.1017/S1355617704105134 15327723

[4] Andreou G Makanikas K. Obstructive Sleep apnoea hypopnea syndrome (OSAS) and its effects on language abilities on Children and elder adult. In: Andreou G (editor) Language and Special Education: Psycholinguistic View. Thessaloniki (2014) p. 185–213. 10.1155/2014/768210 24649370 PMC3932644

[5] Andreou G Vlachos F Makanikas K. Neurocognitive deficits in patients with Obstructive Sleep Apnea Syndrome (OSAS). In: Heinbockel T (editor) Neuroscience. Rijeka (2012) p. 93–115.

[6] Kosmidis MH Vlahou C Panagiotaki P. The verbal fluency task in the Greek population: Normative date, and clustering and switching strategies. J Int Neuropsychol Soc. (2004) 10:164–72. 10.1017/S1355617704102014 15012836

[7] Lim W Bardwell WA Loredo JS Kim EJ Ancoli-Israel S Morgan EE. Neuropsychological effects of 2-week continuous positive airway pressure treatment and supplemental oxygen in patients with obstructive sleep apnea: a randomized placebo-controlled study. J Clin Sleep Med. (2007) 3:380–6. 10.5664/jcsm.26860 17694727 PMC1978310

[8] Ferini-Strambi L Baietto C Di Gioia MR Castaldi P Castronovo C Zucconi M. Cognitive dysfunction in patients with obstructive sleep apnea (OSA): partial reversibility after continuous positive airway pressure (CPAP). Brain Res Bull. (2003) 61:87–92. 10.1016/S0361-9230(03)00068-6 12788211

[9] Twigg GL Papaioannou I Jackson M Ghiassi R Shaikh Z Jaye J. Obstructive sleep apnea syndrome is associated with deficits in verbal but not visual memory. Am J Respir Critical Care Med. (2010) 182:98–103. 10.1164/rccm.200901-0065OC 20299536 PMC2902762

[10] Torelli F Moscufo N Garreffa G Placidi F Romigi A Zannino S. Cognitive profile and brain morphological changes in obstructive sleep apnea. NeuroImage. (2011) 54:787–93. 10.1016/j.neuroimage.2010.09.065 20888921 PMC4169712

[11] Monasterio C Vidal S Duran J Ferrer M Carmona C Barbe F. Effectiveness of continuous positive airway pressure in mild sleep apnea-hypopnea syndrome. Am J Respir Crit Care Med. (2001) 164:939–43. 10.1164/ajrccm.164.6.2008010 11587974

[12] Naëgelé B Thouvard V Pépin JL Lévy P Bonnet C Perret JE. Deficits of cognitive executive function in patients with sleep apnea syndrome. Sleep. (1995) 18:43–52. 7761742

[13] Valencia-Flores M Bliwise D Guilleminault C Cilveti R Clerk A. Cognitive function in patient with sleep apnea after acute nocturnal nasal continuous positive airway pressure (CPAP) treatment: sleepiness and hypoxemia effects. J Clin Exp Neuropsychol. (1996) 18:197–210. 10.1080/01688639608408275 8780955

[14] Verstraeten E. Neurocognitive effects of obstructive sleep apnea syndrome. Curr Neurol Neurosci Rep. (2007) 7:161–6. 10.1007/s11910-007-0012-8 17324368

[15] Shallice T. From neuropsychology to mental structure. Cambridge University Press, New York (1988). 10.1017/CBO9780511526817 17748711

[16] Elias MF Elias PK Robbins MA Wolf PA D'Agostino RB. Cardiovascular risk factors and cognitive functioning: An epidemiological perspective. In: Waldstein SR, Elias MF (eds) Neuropsychology of Cardiovascular Disease. (2001). p. 83–104.

[17] Johns MWA. new method for measuring daytime sleepiness: the Epworth sleepiness scale. Sleep. (1991) 14:540–5. 10.1093/sleep/14.6.540 1798888

[18] Simos P Kasselimis D Mouzaki A. Adult norms for vocabulary measures in Greek: II. Derivation of short forms and effects of health status. Aphasiology. (2009) 25:1–19. 10.1080/02687030802480478

[19] Ross TP Calhoun E Cox T Wenner C Kono W Pleasant M. The reliability and validity of qualitative scores for the controlled oral word association test. Arch Clin Neuropsychol. (2007) 22:475–88. 10.1016/j.acn.2007.01.026 17317094

[20] Rechtschaffen A Kales A. A Manual of Standardized Terminology, Techniques and Scoring System for Sleep Stages of Human Subjects. Public Health Service, US Government Printing Office, Washington DC. (1968).11422885

[21] American Sleep Disorders Association. EEG arousals: scoring rules and examples. Sleep. (1992) 15:173–84. 10.1093/sleep/15.2.173 11032543

[22] Hayes AF Scharkow M. The relative trustworthiness of inferential tests of the indirect effect in statistical mediation analysis: does method really matter? Psychol Sci. (2013) 24:1918–27. 10.1177/0956797613480187 23955356

[23] Lloberes P Durán-Cantolla J Martínez-García MÁ Marín JM Ferrer A Corral J. Diagnosis and treatment of sleep apnea-hypopnea syndrome. Arch Bronconeumol. (2011) 47:143–56. 10.1016/S1579-2129(11)70034-9 21398016

[24] Oveisgharan S Hachinski V. Hypertension, executive dysfunction, and progression to dementia: the Canadian study of health and aging. Arch Neurol. (2010) 67:187–92. 10.1001/archneurol.2009.312 20142526

[25] Wiberg B Lind L Kilander L Zethelius B Sundelöf JE Sundström J. Cognitive function and risk of stroke in elderly men. Neurology. (2010) 74:379–85. 10.1212/WNL.0b013e3181ccc516 20124202

[26] Glynn RJ Beckett LA Hebert LE Morris MC Scherr PA Evans DA. Current and remote blood pressure and cognitive decline. JAMA. (1999) 281:438–45. 10.1001/jama.281.5.438 9952204

[27] Leritz EC McGlinchey RE Kellison I Rudolph JL Milberg WP. Cardiovascular disease risk factors and cognition in the elderly. Curr Cardiovasc Risk Rep. (2011) 5:407–12. 10.1007/s12170-011-0189-x 22199992 PMC3245189

[28] Takeda J Matos TM de Souza-Talarico JN. Cardiovascular risk factors and cognitive performance in aging. Dementia & Neuropsychologia. (2017) 11:442–8. 10.1590/1980-57642016dn11-040015 29354226 PMC5770004

[29] Knopman D Boland LL Mosley T Howard G Liao D Szklo M. Cardiovascular risk factors and cognitive decline in middle-aged adults. Neurology. (2001) 56:42–8. 10.1212/WNL.56.1.42 11148234

[30] Hebert LE Scherr PA Bennett DA Bienias JL Wilson RS Morris MC. Blood pressure and late-life cognitive function change: a biracial longitudinal population study. Neurology. (2004) 62:2021–4. 10.1212/01.WNL.0000129258.93137.4B 15184608

[31] Logroscino G Kang JH Grodstein F. Prospective study of type 2 diabetes and cognitive decline in women aged 70–81 years. Br Med J. (2004) 328:548. 10.1136/bmj.37977.495729.EE 14980984 PMC381043

[32] Roebuck-Spencer TM Glen T Puente AE Denney RL Ruff RM Hostetter G. Cognitive screening tests versus comprehensive neuropsychological test batteries: a national academy of neuropsychology education paper. Arch Clin Neuropsychol. (2017) 32:491–8. 10.1093/arclin/acx021 28334244

